# Supplementary material for: A consumer emotion analysis system based on support vector regression model
Source: PeerJ Comput Sci. 2023 May 9;9:e1381. doi: 10.7717/peerj-cs.1381 (PMC10280612; doi:10.7717/peerj-cs.1381)
Supplement: Supplemental Information 2 [file peerj-cs-09-1381-s002.py]

RCNN/preprocessing\_RCNN.py at 4530523c6ae1108db622bd4f9ea76e0ae10fd6a5 · yangxue0827/RCNN · GitHub


Skip to content


Toggle navigation

Sign up

- Product

  - Actions

    Automate any workflow
  - Packages

    Host and manage packages
  - Security

    Find and fix vulnerabilities
  - Codespaces

    Instant dev environments
  - Copilot

    Write better code with AI
  - Code review

    Manage code changes
  - Issues

    Plan and track work
  - Discussions

    Collaborate outside of code
  - Explore
  - All features
  - Documentation
  - GitHub Skills
  - Blog
- Solutions

  - For
  - Enterprise
  - Teams
  - Startups
  - Education
  - By Solution
  - CI/CD & Automation
  - DevOps
  - DevSecOps
  - Case Studies
  - Customer Stories
  - Resources
- Open Source

  - GitHub Sponsors

    Fund open source developers
  - The ReadME Project

    GitHub community articles
  - Repositories
  - Topics
  - Trending
  - Collections
- Pricing

- In this repository

  All GitHub
  ↵

  Jump to
  ↵

- No suggested jump to results

- In this repository

  All GitHub
  ↵

  Jump to
  ↵
- In this user

  All GitHub
  ↵

  Jump to
  ↵
- In this repository

  All GitHub
  ↵

  Jump to
  ↵

Sign in

Sign up

{{ message }}

yangxue0827
/
**RCNN**
Public

- Notifications
- Fork
  89
- Star
   169

- Code
- Issues
  7
- Pull requests
  0
- Actions
- Projects
  0
- Security
- Insights

More


- Code
- Issues
- Pull requests
- Actions
- Projects
- Security
- Insights

Permalink

4530523c6a

Switch branches/tags


Branches
Tags

Could not load branches


Nothing to show

{{ refName }}
default
View all branches

Could not load tags


Nothing to show


{{ refName }}
default
View all tags

# Name already in use

A tag already exists with the provided branch name. Many Git commands accept both tag and branch names, so creating this branch may cause unexpected behavior. Are you sure you want to create this branch?

 Cancel
 Create

## RCNN/**preprocessing\_RCNN.py** / Jump to Code definitions resize\_image Function if\_intersection Function IOU Function clip\_pic Function load\_train\_proposals Function load\_from\_npy Function Code navigation index up-to-date

 Go to file

 

- Go to file
  T
- Go to line
  L
- Go to definition
  R
- Copy path
- Copy permalink

This commit does not belong to any branch on this repository, and may belong to a fork outside of the repository.

yangxue0827

Update preprocessing\_RCNN.py

Latest commit
79daeee
Sep 5, 2017


**History**

**1**
contributor


### Users who have contributed to this file

143 lines (131 sloc)
5.11 KB

Raw
  Blame

Edit this file

E

 Open in GitHub Desktop

- View raw
- Copy raw contents
   Copy raw contents

   Copy raw contents

   Copy raw contents
- View blame

This file contains bidirectional Unicode text that may be interpreted or compiled differently than what appears below. To review, open the file in an editor that reveals hidden Unicode characters.
Learn more about bidirectional Unicode characters

Show hidden characters


|  |  |
| --- | --- |
|  | from \_\_future\_\_ import division, print\_function, absolute\_import |
|  | import numpy as np |
|  | import selectivesearch |
|  | import tools |
|  | import cv2 |
|  | import config |
|  | import os |
|  | import random |
|  |  |
|  |  |
|  | def resize\_image(in\_image, new\_width, new\_height, out\_image=None, resize\_mode=cv2.INTER\_CUBIC): |
|  | img = cv2.resize(in\_image, (new\_width, new\_height), resize\_mode) |
|  | if out\_image: |
|  | cv2.imwrite(out\_image, img) |
|  | return img |
|  |  |
|  |  |
|  | # IOU Part 1 |
|  | def if\_intersection(xmin\_a, xmax\_a, ymin\_a, ymax\_a, xmin\_b, xmax\_b, ymin\_b, ymax\_b): |
|  | if\_intersect = False |
|  | if xmin\_a < xmax\_b <= xmax\_a and (ymin\_a < ymax\_b <= ymax\_a or ymin\_a <= ymin\_b < ymax\_a): |
|  | if\_intersect = True |
|  | elif xmin\_a <= xmin\_b < xmax\_a and (ymin\_a < ymax\_b <= ymax\_a or ymin\_a <= ymin\_b < ymax\_a): |
|  | if\_intersect = True |
|  | elif xmin\_b < xmax\_a <= xmax\_b and (ymin\_b < ymax\_a <= ymax\_b or ymin\_b <= ymin\_a < ymax\_b): |
|  | if\_intersect = True |
|  | elif xmin\_b <= xmin\_a < xmax\_b and (ymin\_b < ymax\_a <= ymax\_b or ymin\_b <= ymin\_a < ymax\_b): |
|  | if\_intersect = True |
|  | else: |
|  | return if\_intersect |
|  | if if\_intersect: |
|  | x\_sorted\_list = sorted([xmin\_a, xmax\_a, xmin\_b, xmax\_b]) |
|  | y\_sorted\_list = sorted([ymin\_a, ymax\_a, ymin\_b, ymax\_b]) |
|  | x\_intersect\_w = x\_sorted\_list[2] - x\_sorted\_list[1] |
|  | y\_intersect\_h = y\_sorted\_list[2] - y\_sorted\_list[1] |
|  | area\_inter = x\_intersect\_w \* y\_intersect\_h |
|  | return area\_inter |
|  |  |
|  |  |
|  | # IOU Part 2 |
|  | def IOU(ver1, vertice2): |
|  | # vertices in four points |
|  | vertice1 = [ver1[0], ver1[1], ver1[0]+ver1[2], ver1[1]+ver1[3]] |
|  | area\_inter = if\_intersection(vertice1[0], vertice1[2], vertice1[1], vertice1[3], vertice2[0], vertice2[2], vertice2[1], vertice2[3]) |
|  | if area\_inter: |
|  | area\_1 = ver1[2] \* ver1[3] |
|  | area\_2 = vertice2[4] \* vertice2[5] |
|  | iou = float(area\_inter) / (area\_1 + area\_2 - area\_inter) |
|  | return iou |
|  | return False |
|  |  |
|  |  |
|  | # Clip Image |
|  | def clip\_pic(img, rect): |
|  | x = rect[0] |
|  | y = rect[1] |
|  | w = rect[2] |
|  | h = rect[3] |
|  | x\_1 = x + w |
|  | y\_1 = y + h |
|  | # return img[x:x\_1, y:y\_1, :], [x, y, x\_1, y\_1, w, h] |
|  | return img[y:y\_1, x:x\_1, :], [x, y, x\_1, y\_1, w, h] |
|  |  |
|  |  |
|  | # Read in data and save data for Alexnet |
|  | def load\_train\_proposals(datafile, num\_clss, save\_path, threshold=0.5, is\_svm=False, save=False): |
|  | fr = open(datafile, 'r') |
|  | train\_list = fr.readlines() |
|  | # random.shuffle(train\_list) |
|  | for num, line in enumerate(train\_list): |
|  | labels = [] |
|  | images = [] |
|  | tmp = line.strip().split(' ') |
|  | # tmp0 = image address |
|  | # tmp1 = label |
|  | # tmp2 = rectangle vertices |
|  | img = cv2.imread(tmp[0]) |
|  | img\_lbl, regions = selectivesearch.selective\_search( |
|  | img, scale=500, sigma=0.9, min\_size=10) |
|  | candidates = set() |
|  | for r in regions: |
|  | # excluding same rectangle (with different segments) |
|  | if r['rect'] in candidates: |
|  | continue |
|  | # excluding small regions |
|  | if r['size'] < 220: |
|  | continue |
|  | if (r['rect'][2] \* r['rect'][3]) < 500: |
|  | continue |
|  | # resize to 227 \* 227 for input |
|  | proposal\_img, proposal\_vertice = clip\_pic(img, r['rect']) |
|  | # Delete Empty array |
|  | if len(proposal\_img) == 0: |
|  | continue |
|  | # Ignore things contain 0 or not C contiguous array |
|  | x, y, w, h = r['rect'] |
|  | if w == 0 or h == 0: |
|  | continue |
|  | # Check if any 0-dimension exist |
|  | [a, b, c] = np.shape(proposal\_img) |
|  | if a == 0 or b == 0 or c == 0: |
|  | continue |
|  | resized\_proposal\_img = resize\_image(proposal\_img, config.IMAGE\_SIZE, config.IMAGE\_SIZE) |
|  | candidates.add(r['rect']) |
|  | img\_float = np.asarray(resized\_proposal\_img, dtype="float32") |
|  | images.append(img\_float) |
|  | # IOU |
|  | ref\_rect = tmp[2].split(',') |
|  | ref\_rect\_int = [int(i) for i in ref\_rect] |
|  | iou\_val = IOU(ref\_rect\_int, proposal\_vertice) |
|  | # labels, let 0 represent default class, which is background |
|  | index = int(tmp[1]) |
|  | if is\_svm: |
|  | if iou\_val < threshold: |
|  | labels.append(0) |
|  | else: |
|  | labels.append(index) |
|  | else: |
|  | label = np.zeros(num\_clss + 1) |
|  | if iou\_val < threshold: |
|  | label[0] = 1 |
|  | else: |
|  | label[index] = 1 |
|  | labels.append(label) |
|  | tools.view\_bar("processing image of %s" % datafile.split('\\')[-1].strip(), num + 1, len(train\_list)) |
|  | if save: |
|  | np.save((os.path.join(save\_path, tmp[0].split('/')[-1].split('.')[0].strip()) + '\_data.npy'), [images, labels]) |
|  | print(' ') |
|  | fr.close() |
|  |  |
|  |  |
|  | # load data |
|  | def load\_from\_npy(data\_set): |
|  | images, labels = [], [] |
|  | data\_list = os.listdir(data\_set) |
|  | # random.shuffle(data\_list) |
|  | for ind, d in enumerate(data\_list): |
|  | i, l = np.load(os.path.join(data\_set, d)) |
|  | images.extend(i) |
|  | labels.extend(l) |
|  | tools.view\_bar("load data of %s" % d, ind + 1, len(data\_list)) |
|  | print(' ') |
|  | return images, labels |

- Copy lines
- Copy permalink
- View git blame
- Reference in new issue

 Go

## Footer

© 2023 GitHub, Inc.

### Footer navigation

- Terms
- Privacy
- Security
- Status
- Docs
- Contact GitHub
- Pricing
- API
- Training
- Blog
- About

You can’t perform that action at this time.

You signed in with another tab or window. Reload to refresh your session.
You signed out in another tab or window. Reload to refresh your session.
